# Human Immunodeficiency Virus Type 1 Counseling and Testing Program in the Prenatal Setting

**DOI:** 10.1155/S106474499500069X

**Published:** 1995

**Authors:** Arlene D. Bardeguez, Thomas Denny, Paul Palumbo, Yvonne Wesley, James Oleske, Edward Connor, Gerson Weiss

**Affiliations:** 1 Department of Obstetrics and Gynecology University of Medicine and Dentistry-New Jersey Medical School Newark NJ USA; 2 AIDS Clinical Trials Unit University of Medicine and Dentistry-New Jersey Medical School Newark NJ USA; 3 Department of Pediatrics University of Medicine and Dentistry-New Jersey Medical School and Children's Hospital of New Jersey Newark NJ USA; 4 Department of Obstetrics and Gynecology New Jersey Medical School 185 South Orange Avenue MSB E-506 Newark NJ 07103 USA

## Abstract

*Objective:* The objectives of this study were to ascertain the acceptance rate of human immunodeficiency virus type 1 (HIV-1) testing in a high-prevalence area and to describe the sociodemographic and clinical characteristics of seropositive women diagnosed in the prenatal setting.

*Methods:* A retrospective review was carried out of the prenatal HIV-1 counseling and testing program at University Hospital, Newark, NJ (1989-1990).

Results: Sixty-seven percent (741/1,114) of the women offered HIV-1 counseling services accepted testing and 40 (40/741:5.3%) new cases were identified. Heterosexual contact was the primary exposure (17:52%) of these women, of whom 13 (73%) had negative syphilis serologies. Sixty-four percent were asymptomatic. The mean absolute CD4 lymphocyte count in seropositive women was 514 ± 305 cells/mm^3^ . Severe immunosuppression was seen in 7/32 (22%) patients. Seventy-three percent (24/33) depended on public-assistance programs for their health-care services.

*Conclusions:* A voluntary HIV-1 counseling and testing program is well accepted in the prenatal setting. It can provide early identification of asymptomatic seropositive women and infants at risk and lead to early intervention and therapy.


Infectious Diseases in Obstetrics and Gynecology 3:229–235 (1995)
© 1996 Wiley-Liss, Inc.

### Human Immunodeficiency Virus Type 1 Counseling and Testing Program in the Prenatal Setting

**Arlene D. Bardeguez, Thomas Denny, Paul Palumbo, Yvonne Wesley, James Oleske, Edward Connor, and Gerson Weiss**

*Department of Obstetrics and Gynecology (A.D.B., G.W.) and AIDS Clinical Trials Unit (A.D.B., T.D., P.P., Y.W., J.O., E.C.), University of Medicine and Dentistry-New Jersey Medical School, and Department of Pediatrics, University of Medicine and Dentistry-New Jersey Medical School and Children's Hospital of New Jersey (T.D., P.P., Y.W., J.O., E.C.), Newark, NJ*

### Abstract

***Objective:*** **The objectives of this study were to ascertain the acceptance rate of human immunodeficiency virus type 1 (HIV-1) testing in a high-prevalence area and to describe the sociodemographic and clinical characteristics of seropositive women diagnosed in the prenatal setting.**

***Methods:*** **A retrospective review was carried out of the prenatal HIV-1 counseling and testing program at University Hospital, Newark, NJ (1989–1990).**

***Results:*** **Sixty-seven percent (741/1,114) of the women offered HIV-1 counseling services accepted testing and 40 (40/741:5.3%) new cases were identified. Heterosexual contact was the primary exposure (17:52%) of these women, of whom 13 (73%) had negative syphilis serologies. Sixty-four percent were asymptomatic. The mean absolute CD4 lymphocyte count in seropositive women was 514 ± 305 cells/mm$^3$. Severe immunosuppression was seen in 7/32 (22%) patients. Seventy-three percent (24/33) depended on public-assistance programs for their health-care services.**

***Conclusions:*** **A voluntary HIV-1 counseling and testing program is well accepted in the prenatal setting. It can provide early identification of asymptomatic seropositive women and infants at risk and lead to early intervention and therapy.** © 1996 Wiley-Liss, Inc.

Key words

HIV-1 seroprevalence, pregnancy, immunosuppression, AIDS

In the United States, the human immunodeficiency virus (HIV)/acquired immunodeficiency syndrome (AIDS) epidemic has increasingly affected women of childbearing age from racial or ethnic minorities living in urban communities.[1-3] HIV/AIDS is a leading cause of mortality for these women.[4] A delay in diagnosis and treatment frequently results in increased morbidity from opportunistic infections such as *Pneumocystis carinii* pneumonia (PCP).[5,6]

The number of women with AIDS as well as the HIV seroprevalence among parturients in New Jersey is higher than the national average.[7,8] In general, more than 80% of the women in the United States seek the health-care system for reproductive decisions including evaluation for sexually transmitted diseases (STDs), family planning, and pregnancy. For example, in 1986, approximately 60% of black and hispanic women and 75–80% of white women initiated their prenatal care during the first trimester of pregnancy.[9] Therefore, the availability of HIV-1 counseling and testing programs in these settings could identify and educate women at risk. In addition, these programs could provide the uninfected woman with knowledge of the modes of HIV transmission, identify risk behavior that may lead

Address correspondence/reprint requests to Dr. Arlene D. Bardeguez, Department of Obstetrics and Gynecology, New Jersey Medical School, 185 South Orange Avenue MSB E-506, Newark, NJ 07103.

Received October 16, 1995
Accepted February 13, 1996

to the acquisition of HIV, and give the option of selecting modalities effective in preventing pregnancy and STDs, including HIV. For the pregnant woman, the early identification of HIV infection provides an opportunity to 1) increase her knowledge of the efficacy and toxicity of current treatment methods; 2) make choices regarding her pregnancy[10,11] and the obstetrical care that minimizes morbidity and mortality of HIV disease;[12,13] 3) initiate strategies to reduce perinatal HIV-1 transmission;[14,15] and 4) provide early HIV diagnosis and treatment of her newborn.[16,17] The pregnant HIV-infected woman could also benefit from participating in clinical trials evaluating the current therapeutic regimens.[18]

Most HIV-1 counseling and testing of women occur at either reproductive-health clinics (family-planning or prenatal clinics), STD clinics, or HIV counseling sites.[19,20] Sweeney et al.[21] found the highest median rate (0.63%) for HIV-1 infection among minority women receiving prenatal services in the United States and Puerto Rico. The lowest median rate (0.21%)[21] was observed among white women attending abortion/family-planning clinics. Women constituted 24% of all newly identified HIV-1 cases in STD clinics in Baltimore.[22] The majority of these women were asymptomatic with absolute CD4 lymphocyte counts of $\geq 200/mm^3$ and intravenous drug use (IVDU) as their primary exposure risk category.[22] Prior studies have not explored the effect of establishing HIV counseling and testing programs in prenatal clinics of HIV-1 prevalence areas of >3/1,000 live births.

The goals of this study were to describe 1) the acceptance rate of HIV-1 testing among women attending a prenatal clinic in a high-prevalence area during the first year of a counseling and testing program, 2) the HIV-1 prevalence among pregnant women attending a public prenatal clinic, and 3) the sociodemographic and clinical characteristics of these newly diagnosed women.

### SUBJECTS AND METHODS

Routine, voluntary HIV-1 counseling and testing were initiated in the prenatal clinic at University Hospital, Newark, NJ, in September 1989. During the first year (September 1989–August 1990) of this program, any pregnant woman willing to accept an individual, private counseling session (which lasted 15–30 min) received HIV-1 counseling as part of her initial prenatal visit. All counseling sessions were provided by HIV counselors certified by the New Jersey Department of Health Program. During these sessions, the counselors provided information regarding risk behavior, reliability of the assay to detect HIV antibody, interpretation of the results, and availability of therapeutic options. At the end of each counseling session, each woman was asked to sign a consent form if she desired HIV testing.

A woman considered at high risk for HIV-1 infection was also offered participation in the prospective Perinatal HIV-1 Transmission Study (PHS) during the counseling session. The factors judged to constitute high risk for HIV-1 infection included past or current substance abuse, partner of an HIV-infected male, partner of a bisexual or an IVDU male, sex worker, recipient of a blood transfusion before 1985, or birth in a pattern II country. An informed consent approved by the Institutional Human Research Committee was signed by each volunteer who elected to participate in the PHS. Each woman enrolled in the PHS had a lymphocyte profile done at her initial visit as part of her research evaluation. The sociodemographic data including a risk assessment were collected. IVDU was considered the mode of exposure for those women with documented prior or current IVDU. Heterosexual transmission was considered the mode of exposure for those women whose partners were HIV infected or whose partners belonged to a high risk category such as IVDU. All samples were analyzed for HIV-1 in an AIDS Clinical Trials Group (ACTG)-certified laboratory or a state-certified laboratory. The test results were given during the post-test counseling sessions. A woman found to be negative was advised to repeat the HIV testing in 6 months, while a woman found to be HIV infected was offered treatment. The HIV-1 positive pregnant women were also referred to appropriate medical and social services.

An HIV-1 enzyme-linked immunosorbent assay (Abbott, Chicago, IL) was used for screening all samples. Persistent positive samples were confirmed by Western blot tests (Dupont, Wilmington, DE). The interpretation of these assays was based on the criteria used by the Association of State Public Health Laboratory Directors/Centers for Disease Control and Prevention (CDC). A lymphocyte subset analysis was done within 6 h of collection, using standard whole-blood lysing methodology in an ACTG-certified laboratory.[23]

The HIV-1 positive patients were assigned to clinical categories (asymptomatic, symptomatic, or AIDS) based on the CDC 1987 revised classification system for HIV infection and disease in adolescents and adults.[24] The patients with constitutional symptoms including fever (38.5°C) or diarrhea lasting >1 month, herpes zoster, pelvic inflammatory disease, thrombocytopenia, or oropharyngeal candidiasis (thrush) were categorized as symptomatic. The patients with HIV encephalopathy, malignancy, or opportunistic infections such as PCP, cytomegalovirus disease, or candidiasis of the esophagus were classified as having AIDS. The patients were also classified using the 1993 expanded AIDS surveillance case definition. This revised system expanded the clinical AIDS category to include invasive cervical cancer, recurrent pneumonia, and *Mycobacterium tuberculosis* infections. In addition, this system categorized patients according to their immune competence within 3 CD4 lymphocyte count strata, namely ≥500, 200–499, and <200 cells/µl. Asymptomatic or symptomatic patients with CD4 lymphocyte counts of <200 cells/µl met the immunologic criteria for the diagnosis of AIDS.[25]

The information regarding the number of new patients attending the ambulatory clinic was obtained from the annual OB/GYN departmental report. The number of patients receiving HIV counseling and screening was documented by the HIV counselors assigned to the prenatal clinic during the study period. The patient information was collected using a standardized data collection form for all participants of the PHS. In addition, health-care resources and risk factors were included. The means and standard deviations were calculated and used to describe the sociodemographic and laboratory parameters of the cohort.

### RESULTS

Between September 1989 and August 1990, 1,114/1,863 (60%) pregnant women attending the ambulatory low-risk prenatal clinic received HIV counseling. Of these, 741 (67%) women accepted HIV testing during the study period. A trend toward an increased acceptance rate for HIV testing over time was noted, with a rise from 53% during the first 3 months to 73% between April and September 1990 ($P = 0.009$) (Fig. 1). Forty new cases of HIV infection were identified (5.3% among women who agreed to be tested). All of these patients were residents of the inner city of Newark and surrounding communities. Thirty-three (83%) HIV-positive pregnant women consented to be followed in the PHS, 3 patients did not receive their serostatus until the postpartum visit, 2 refused HIV-related care, and 2 were lost to follow-up in the prenatal clinic.

Ninety-four percent of the newly identified HIV positive pregnant women belonged to racial or ethnic minority groups. This distribution is representative of the population seen in the prenatal clinic during the study period. The mean age of the seropositive women was 30 ± 6 years, while 10 women were 35 years or older (Fig. 2). The mean gestational age at the time of diagnosis was 26 weeks (median 24 weeks); however, 8 patients received their diagnosis at <20 but >14 weeks gestation. No patient was screened or tested at <14 weeks. Of those who received their diagnoses <20 weeks, none desired an abortion. Two patients (≥35 years old) received genetic counseling and amniocentesis prior to knowledge of their serostatus. Heterosexual transmission was the primary risk category (52%) and IVDU the next highest (48%) Nine (9/33:27%) pregnant women also tested positive for syphilis. Five of these women had IVDU as their exposure risk factor. The selected clinical characteristics of the newly identified cases who received care (N = 33) are listed in Table 1.

The lymphocyte profile at the time of diagnosis was available for 97% (32/33) of the women enrolled in the PHS (Table 2). The clinical HIV-disease stage was assigned at entry and reevaluated 6 months postpartum. No patient was found to have clinical HIV-disease progression. However, when comparing disease stages in this cohort based on the 1987 adult/adolescent CDC classification with the most recent 1993 definition, we found that 22% (7/32) of the previously asymptomatic women met the AIDS definition based on abnormal immune profiles (Fig. 3).[23] The AIDS-defining illnesses seen among the women in this cohort were PCP, AIDS dementia, wasting syndrome, and oroesophageal candidiasis. Overall, 6 patients in this cohort received HIV-related medications during the antepartum period for maternal indications. The drugs used included antiretrovirals (N = 3), PCP prophylaxis (N = 5), and antituberculosis medications (N = 1).

Seventy-three percent (24/33) of the women relied on public-assistance programs for their health

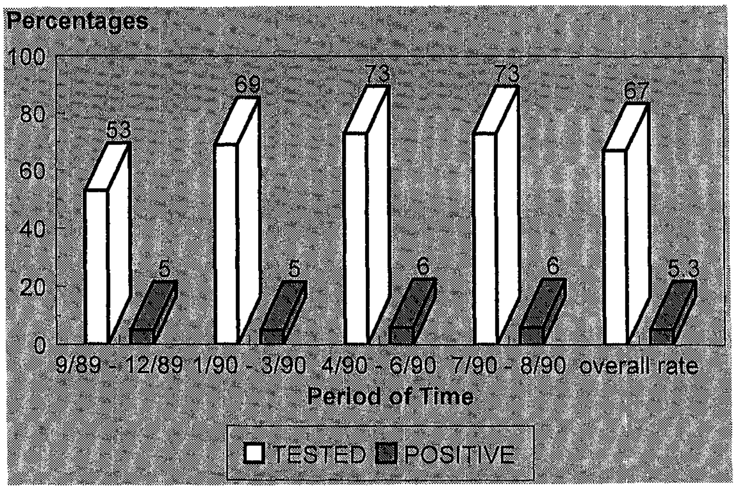

Fig. 1. Trend in acceptance rate (%) of HIV-1 testing and HIV-1 seroprevalence in the prenatal clinic of University Hospital, Newark NJ (September 1989–August 1990).

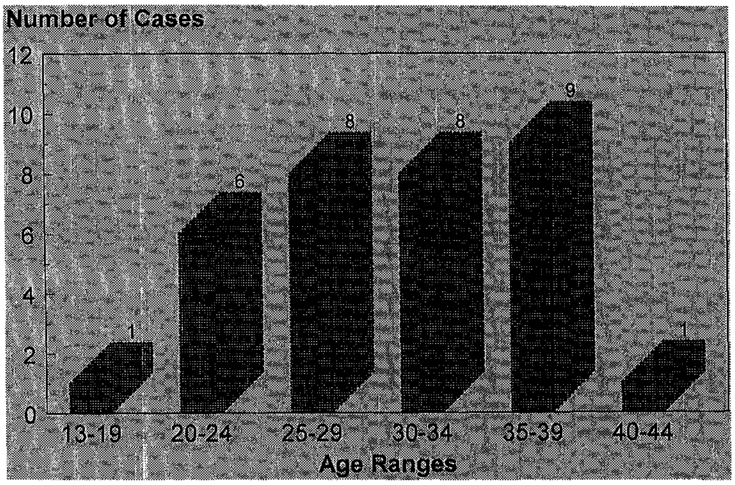

Fig. 2. Age distribution of newly identified cases of HIV-1 infection in pregnant women, University Hospital, Newark, NJ (September 1989–August 1990).

care, 15% (5/33) were covered through their employer or their spouse's employer, and 12% (4/33) had no health-care resources. The most common public-assistance programs used by this population were Welfare, Aid for Families With Dependent Children, and Supplemental Security Income. The mean yearly income for women in the self-pay category (not on public assistance) was $9,156.

### DISCUSSION

The present study shows that 40 new cases of HIV infection were identified during the first year of establishing a counseling and testing program based in the prenatal setting. The HIV-1 incidence seen among childbearing women attending this prenatal clinic was 5.3%. This observation supports the establishment of HIV-1 counseling and testing as a routine component of prenatal care for women living in areas of high prevalence. The increased acceptance of HIV-1 testing over the first year of this program from 53 to 73% ($P = 0.04$) suggests that the prenatal clinic is an optimal setting to reach childbearing-age women. For those women diagnosed as HIV infected, we incorporated their HIV-

TABLE 1. Selective clinical characteristics of newly identified HIV-positive pregnant women

| Characteristic | N |
|---|---|
| Gestational age at time of diagnosis (N = 33) | |
| 1st trimester (<14 weeks) | 0 |
| 2nd trimester (14–27 weeks) | 19 |
| 3rd trimester (≥28 weeks) | 14 |
| Baseline CD4 cells/μl (N = 32) | |
| ≥500 | 17 |
| 201–499 | 8 |
| <200 | 7[a] |
| Syphilis serology (N = 32) | |
| Positive | 9 |
| Negative | 23 |

[a]Diagnosed as AIDS in the 1993 CDC expanded surveillance case definition.

related care in the prenatal setting, decreasing the number of referral visits and availing their infants of early HIV-1 diagnosis and treatment. We believe that these factors, together with the increased experience gained by the counselors in improving patients' knowledge regarding the modes of exposure, prolonged the survival of HIV-infected subjects. We further believe that the availability of HIV-related therapies and appropriate care was responsible for the increased acceptability of the program among patients and health-care workers. Similar programs should be available for women receiving care in a private setting.

Most seropositive women in this study were asymptomatic. In contrast to a report by Hutchinson et al.,[22] the majority (73%) of the pregnant women were negative for syphilis and were not IVDU. These cases would have generally been missed using a risk-assessment approach alone. Of the clinically asymptomatic women, 47% (15/32) had CD4 lymphocyte counts that met the criteria for the initiation of antiretroviral treatment at the time of diagnosis.[12] Furthermore, 22% (7/32) of the women met the criteria for the initiation of PCP prophylaxis.[13] The immunologic compromise of these pregnant women was confirmed by the increase in the number of AIDS cases in the cohort when the 1993 adult/adolescent CDC definition was used (Figure 3). This expanded definition classifies individuals with CD4 lymphocyte counts of <200 cells/mm$^3$ in the AIDS category (A3) surveillance.[25] The increased survival observed among subjects on antiretroviral therapy and PCP prophylaxis and the availability of such drugs through a state-supported program warrant access to HIV testing during pregnancy and postpartum for women of reproductive age.

Women are likely to access the health-care system during pregnancy. Thus, broader availability of HIV counseling and testing services in the prenatal-care setting could identify new cases early in the disease process.[18–22] Efforts to improve access to early prenatal care for under-represented minorities are essential to the success of any HIV counseling and testing program. Ultimately, the long-term success of these programs would require appropriate referrals of HIV-infected women and their infants to local or regional specialized-care networks.

The timely initiation of antiretroviral therapy or PCP prophylaxis can delay the risk of disease progression or prevent the occurrence of common opportunistic infections in immunocompromised pregnant women.[5,12,13] An avoidance of invasive procedures such as amniocentesis, chorionic villi sampling, and fetal blood sampling has been recommended to minimize perinatal transmission.[26] A decrease in the length of ruptured membranes may influence the risk of perinatal transmission.[27] Consequently, the identification of the HIV-infection status in pregnant patients is critical to guide optimal obstetrical practices. A knowledge of the HIV-1 infection status could also benefit the unborn child by allowing the pregnant woman to exercise her option of medical strategies that reduce perinatal HIV transmission, such as the zidovudine regimen used among the participants of ACTG 076.[13,14] The neonates at risk of perinatal HIV infection can also benefit from access to early diagnosis, prophylaxis, and HIV-related therapy, if indicated.[16,17] Lastly, some HIV-infected pregnant women may choose to participate in clinical trials aimed at developing safe and efficacious therapies in pregnancy and reducing perinatal HIV transmission.[14,15]

In our study, we observed a *small* discrepancy between the HIV-1 seroprevalence in this prenatal clinic (5.3%) and the results of the blinded analysis of cord blood in 1987 (4.3%).[28] During the study period, 40% (749/1,863) of the women did not receive HIV counseling, and 33% (373/1,114) refused HIV testing. Consequently, the observed discrepancy could be explained by a lower seroprevalence rate among the patients who were not tested. Monitoring trends of HIV-testing acceptance and HIV seroprevalence over time will be important to clarify

TABLE 2. Lymphocyte profile at the time of diagnosis in pregnant HIV-positive women (N = 32)

| | Immune marker (cells/μl) | | | | |
|---|---|---|---|---|---|
| | CD4 absolute | CD4% | CD8 absolute | CD8% | CD4/CD8 |
| Mean ± SD | 514 ± 305 | 29 ± 13 | 937 ± 402 | 56 ± 12 | 0.57 ± 0.30 |
| Median | 535 | 31 | 881 | 53 | 0.57 |

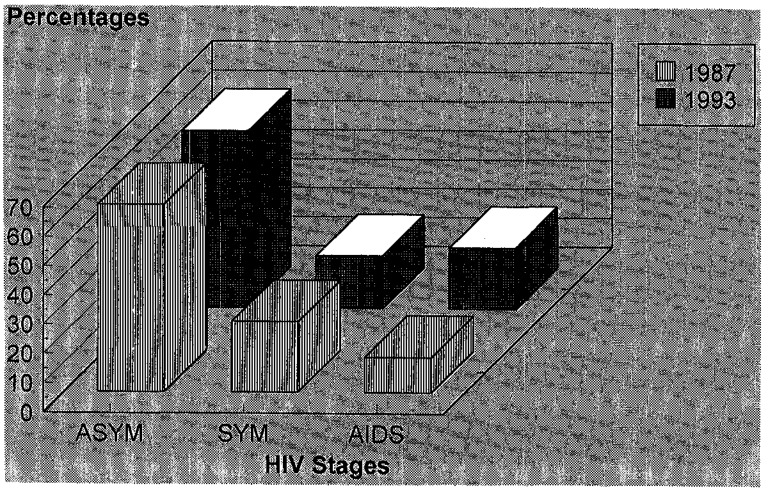

Fig. 3. HIV-1-disease stage of newly identified cases of HIV-1 infection in pregnant women according to 1987 and 1993 CDC adult/adolescent definition. Asym = asymptomatic; Sym = symptomatic.

this issue. Another limitation of our study was that we did not ascertain factors associated with the acceptance of HIV testing during pregnancy. Ethnic or cultural attitudes and beliefs influence the acceptability of HIV testing and HIV-related care. A consideration of these factors might be relevant to achieve the same degree of success when implementing counseling and testing programs in other communities. The benefits gained by the HIV-infected individual and the institution that implements a prenatal HIV counseling and testing program are influenced by the health-care attitudes and beliefs of the members of that community. Therefore, we cannot predict the success of this approach in all communities.

In summary, the knowledge of one's HIV status offers many advantages to the mother and her infant:[5,12–18] an opportunity for education regarding therapy for the mother-infant pair; the option to initiate HIV-related therapy if indicated; the opportunity to discuss potential modifications in antepartum and intrapartum care; the advantage of knowledge regarding the serostatus of her neonate early in the disease process; and the option for elective termination of pregnancy. Although a knowledge of one's HIV status may have a negative impact such as depression, suicide, or risk of battering, the availability of multidisciplinary care and a support infrastructure can palliate the adverse consequences for the mother-infant pair. Our study suggests that routine HIV testing and counseling can be implemented during the prenatal period for women living in areas of high HIV-1 prevalence (>3 cases/1,000 live births). We also described the therapeutic benefit of this approach for the mother and fetus, providing a rationale for implementation of the policies currently recommended by the New Jersey Department of Health, American College of Obstetricians and Gynecologists, and CDC.

### ACKNOWLEDGMENTS

We thank Mini Lester; Cora Leus, R.N.; Michelle Badger, B.A.; and the Newark Perinatal Research staff for their continuous commitment to our mission. We also thank the attendants, residents, nurses, midwives, and other health-care providers of the ambulatory clinics of the Department of Obstetrics and Gynecology for their assistance and support in the implementation of this program. This work was supported by USAMRDC 70090001 from the U.S. Department of Defense, ACTG 125883-01 from the National Institute of Allergy and Infectious Diseases, and U62-CCU203006 from the cooperative agreement U.S. Center for Disease Control and Prevention/Division of Sexually Transmitted Diseases and HIV Prevention.

### REFERENCES

1. Update: Acquired immunodeficiency syndrome—United States, 1992. MMWR 42(28):547–557, 1993.
2. Selik RM, Chu SY, Buehler JW: HIV Infection as leading cause of death among young adults in U.S. cities and states. JAMA 269:2991–2994, 1993.
3. Wasser WC, Gwinn M, Fleming P: Urban-non urban distribution of HIV infection in childbearing women in the United States. J Acquired Immune Deficiency Synd 6:1035–1042, 1993.
4. Update: Mortality attributable to HIV infection/AIDS among persons aged 25–44 years—United States, 1990–1991. MMWR 42:481–486, 1993.
5. Bastian L, Bennett CL, Adams J, Waskin H, et al.: Differences between men and women with HIV-related HIV-1 *Pneumocystis carinii* pneumonia: Experience from 3,000 cases in New York City in 1987. J Acquired Immune Deficiency Synd 6:617–623, 1993.
6. Minkoff HL, deRegt RH, Landesman S, et al.: *Pneumocystis carinii* pneumonia associated with acquired immunodeficiency syndrome in pregnancy: A report of three maternal deaths. Obstet Gynecol 67:284–287, 1986.
7. New Jersey Department of Health: HIV/AIDS Update. Division of AIDS Prevention and Control. November–December 1991.
8. Gwinn M, Pappaioanou M, George R, Hannon H, et al.: Prevalence of HIV infection in childbearing women in the United States. Surveillance using newborn blood samples. JAMA 265:1704–1708, 1991.
9. U.S. Department of Health and Human Services: Health status of the disadvantaged. Chart Book 1990. Public Health Service. Health Resources and Services Administration. Bureau of Health Professions. Division of Disadvantaged Assistance. DHHS Pub. No. (HRSA) HRS-P-DV 90-1 Chapter 4, pp 67–73.
10. Selwyn PA, Carter RJ, Schoenbaum EE, et al.: Knowledge of HIV antibody status and decision to continue or terminate pregnancy among intravenous drug users. JAMA 261:3567–3571, 1989.
11. Childbearing and contraceptive-use plans among women at high risk for HIV infection—Selected U.S. sites, 1989–1991. MMWR 41:135–144, 1992.
12. Sande MA, Carpenter CCJ, Cobbs CG, Holmes KK, et al.: Antiretroviral therapy for adult HIV-infected patients. Recommendations from a state-of-the art conference. JAMA 270:2583–2589, 1993.
13. Sperling RS, Stratton P, and Obstetric-Gynecologic Working Group of the AIDS Clinical Trials Group and the NIAID: Treatment options for human immunodeficiency virus-infected pregnant women. Obstet Gynecol 79:443, 1992.
14. Centers for Disease Control and Prevention: Zidovudine for the prevention of HIV transmission from mother to infant. MMWR 43:285–287, 1994.
15. Recommendations of the U.S. Public Health Service Task Force on the use of zidovudine to reduce perinatal transmission of human immunodeficiency virus. MMWR 43(RR-11):1–20, 1994.
16. Rossi P, et al.: Early diagnosis of HIV infection in infants: Report of a consensus workshop, Sienna, Italy, January 17–18, 1992. J Acquired Immune Deficiency Synd 5:1169–1178, 1992.
17. Simonds RJ, Oxtoby MJ, Caldwell MB, Gwinn ML, Rogers MF: *Pneumocystis carinii* pneumonia among U.S. children with perinatally acquired HIV infection. JAMA 270:470–473, 1993.
18. Lindsay MK: A protocol for routine voluntary antepartum human immunodeficiency virus antibody screening. Am J Obstet Gynecol 168:476–479, 1993.
19. Publicly funded HIV counseling and testing—United States, 1985–1989. MMWR 39:137–152, 1990.
20. Characteristics of HIV infection among women served by publicly funded HIV counseling and testing services—United States, 1989–1990. MMWR 195–204, 1991.
21. Sweeney PA, Onorato IM, Allen D, Byers RH, et al.: Sentinel surveillance of human immunodeficiency virus infection in women seeking reproductive health services in the United States, 1988–1989. Obstet Gynecol 79:503–510, 1992.
22. Hutchinson CM, Wilson C, Reichart CA, Marsoglia VC, et al.: CD4 lymphocyte concentrations in patients with newly identified HIV infection attending STD clinics. JAMA 253–256, 1991.
23. Calvelli T, Denny TN, Paxton H, Gelman R, Kagan JM: Guidelines for flow cytometry immunophenotyping. A report from the National Institute of Allergy and Infectious Diseases/Division of AIDS. Cytometry 14:702–715, 1993.
24. Centers for Disease Control: Revision of the CDC surveillance case definition for acquired immunodeficiency syndrome. MMWR 36(Suppl 1):S3–15, 1987.
25. 1993 revised classification system for HIV infection and expanded surveillance case definition for AIDS among adolescents and adults. MMWR 41:RR-17, 1992.
26. Cotton D, Watts H: Management of HIV infection during pregnancy. AIDS Clin Care 7(6):45–49, 1995.
27. Burns D, Landesman S, Muenz L, et al.: Cigarette smoking, premature rupture of membranes, and vertical transmission of HIV-1 among women with low CD4+ levels. J Acquired Immune Deficiency Synd 7:718–726, 1994.
28. Shapiro CN, Schultz SL, Lee NC, Dondero TJ: Review of the human immunodeficiency virus infection in women in the U.S. Obstet Gynecol 74:800–808, 1989.
